# CAN THE PUBLIC LONG-TERM CARE INSURANCE SERVICES IN JAPAN PREVENT THE DETERIORATION OF CARE LEVELS?

**DOI:** 10.1093/geroni/igac059.2585

**Published:** 2022-12-20

**Authors:** Aki Sugiura, Reiko Kanaya, Yasushi Takeya, Hiroshi Toki, Ryohei Yamamoto, Miyae Yamakawa

**Affiliations:** Osaka University, Suita, Osaka, Japan; Osaka University, Osaka, Osaka, Japan; Osaka University, Suita, Osaka, Japan; Health and Counseling Center, Suita, Osaka, Japan; Osaka University, Suita, Osaka, Japan; Osaka University, Suita, Osaka, Japan

## Abstract

In Japan, the public long-term care insurance system supports a super-aged society. The long-term care service (LTCS) system is designed to prevent deterioration of care levels. We conducted a retrospective cohort study to clarify whether using LTCS prevents the deterioration of care levels. We use anonymized data of the Osaka National Health Insurance Database. We analyzed 16,469 subjects who were 65–74 years of age and certified at the mild care level from 2012 to 2015 at the time of initial certification. A baseline measurement was conducted 1 year after initial certification, and observation was continued until the subject’s care level deteriorated. Statistical analysis was performed using the Kaplan-Meier method and the Cox proportional hazards model. The results revealed that the period until the deterioration of care level (which occurred in 25% of subjects) was 6 months in the service use group and 22 months in the service non-use group. Although we included sex, care level, dementia, and Parkinson’s disease as variables in the statistical analysis, service users were more likely to deteriorate than non-service users (Hazard Risks: 1.89; 95% confidence interval: 1.79–2.00). It is likely that other factors, such as heart disease, may also be involved, and there may be a bias related to family caregiver factors. In conclusion, the current findings suggest that using LTCS cannot prevent the deterioration of care levels. Future studies should verify the effectiveness of LTCS by adding further variables and data regarding family caregivers and changes in outcomes.

